# Outside the healthy bounds: reference intervals for population and individual-level medical speech analysis

**DOI:** 10.3389/fdgth.2026.1870061

**Published:** 2026-08-19

**Authors:** Pascal Hecker, Monica Gonzalez-Machorro, Catarina Botelho, Helly Hammer, Hesam Sagha, Matthias Kahlau, Robert Hoepner, Florian Eyben, Björn W. Schuller, Bert Arnrich

**Affiliations:** 1 Digital Health—Connected Healthcare, Hasso Plattner Institute, University of Potsdam, Potsdam, Germany; 2 audEERING GmbH, Gilching, Germany; 3 CHI—Chair of Health Informatics, TUM University Hospital, Munich, Germany; 4 HLT—Human Language Technologies Lab, INESC-ID, Lisbon, Portugal; 5 Department of Neurology, Inselspital and University of Bern, Bern, Switzerland; 6 GLAM—Group on Language, Audio, & Music, Imperial College London, London, United Kingdom

**Keywords:** artificial intelligence – AI, computational paralinguistics, digital health (e-health), noise robustness, personalised healthcare, reference intervals (RIs), speech analysis and processing, speech biomarkers

## Abstract

Reference intervals (RIs) are a well-established clinical tool for defining healthy bounds of laboratory measures. Recently, they have been applied to acoustic features extracted from speech to provide a transparent and disorder-agnostic perspective on symptomatic speakers. We evaluate the potential and limitations of RIs across three datasets: clean, laboratory recordings from people with multiple sclerosis (pwMS) and two noisy datasets targeting respiratory symptoms and work-related stress, collected under everyday-life conditions. At the population level, we compare the proportion of features outside the RI bounds between control and symptomatic groups, test noise augmentation of the RI-estimation corpus for improved robustness, and identify the most indicative acoustic features for each health condition. At the individual level, we derive a per-speaker deviation score that quantifies how far each speaker’s acoustic profile deviates from the healthy reference, and assess its discriminative ability using the area under the receiver operating characteristic curve (AUC). Noise augmentation of the healthy reference corpus yields similar population-level separation; the continuous ${\mathrm{DS}}_{Q123}$ deviation score yielded 12 features that remained significant after global Benjamini–Hochberg false discovery rate (FDR) correction. The individual-level deviation score achieves promising discrimination for male pwMS (AUC=0.97 for read speech), even in a clinically mild cohort, while results for audRespire and the work-related stress dataset remain limited at both levels. RIs show promise as interpretable, disorder-agnostic markers for speech-based health assessment; the individual-level deviation score complements the population-level analysis by providing a per-speaker health indicator that can surface deviations masked in group-level comparisons.

## 1 Introduction

A person’s speech has been shown to be affected by various health-related factors. Due to its wide availability nowadays, there is great promise to harness speech for early onset detection of symptoms, fine-grained tracking of disorder progression, or measuring wellbeing-related indicators (1). Machine learning methods are readily used to quantify the subtleties in speech and to enable these use cases. In the healthcare context, interpretability is key, since practitioners and users both want to comprehend results and potential diagnoses (2). One shortcoming of existing machine learning applications is that they usually only regard a limited set of classes and disorders. However, often, various disorders co-occur and affect each other (3).

Recently, Botelho et al. (3, 4) introduced the concept of defining reference intervals (RIs) for interpretable acoustic features, aiming to characterise speech in a transparent and disorder-agnostic manner. RIs represent the typical range of values for a given parameter within a reference population, commonly defined as the interval between the 2.5th and 97.5th percentiles of the parameter distribution. In this context, an unseen healthy individual would be expected to exhibit speech characteristics that fall within these bounds, provided they belong to a comparable population.

While RIs are well-established in medicine, their application to speech research is novel. In Botelho et al. (3), the authors computed RIs using the Crowdsourced Language Assessment Corpus (CLAC), and based on these intervals, speech from individuals with Parkinson’s disease [PC-GITA (5)] and Alzheimer’s disease [ADReSS (6)] was assessed. More recently, White et al. (7) compared three datasets for deriving RIs, assessed their overlap, and applied these references to patients with major depressive disorder. They reported that RIs across datasets overlapped by approximately 90%, highlighting both potential differences in recording conditions and variability in dataset consistency.

In this work, we extend this approach by deriving RIs with CLAC and applying it on three independent datasets, each targeting a distinct health-related condition: COMMITMENT for multiple sclerosis (MS) (8, 9), Mental Wellbeing at Sea (MWaS) (10, 11), and respiratory symptoms (audRespire) (12). These datasets provide a strong testbed for the reference intervals, as they were recorded under diverse conditions and encompass a broad spectrum of health states. These datasets were all collected with high comparability in mind; in particular, speech tasks were designed to be similar across all these different use cases. Building on the findings of White et al. (7), who reported substantial variation in reference ranges across datasets, we further investigate how noise levels affect the stability of reference intervals and how this, in turn, may impact their use in comparisons with clinical datasets.

While the RI-based studies above assess speech at the population level—comparing group-wise feature distributions between symptomatic and control cohorts—personalised healthcare applications ultimately require methods that can profile individual speakers. RIs lend themselves naturally to such individual-level assessment: by quantifying how each speaker’s features deviate from healthy bounds, one can derive a composite deviation score that provides an interpretable, per-speaker health indicator. Beyond this population-level analysis, we therefore extend the RI framework to an individual-level assessment by deriving per-speaker deviation scores and their discriminative ability across the three probe datasets.

Our main objective is to examine the potential of reference intervals (RIs) as a framework for characterising healthy speech and comparing it across different health states and recording conditions. Specifically, we address 3 research questions: (1) Can noise augmentation be effectively used to adapt the RI framework to noisy, real-world datasets? (2) How do the RIs compare with the controls and the symptomatic individuals included in the COMMITMENT, Mental Wellbeing at Sea, and audRespire datasets, and which acoustic features emerge as discriminative indicators of specific health states within the RI framework? (3) Can individual-level deviation profiles derived from the RI framework discriminate symptomatic from control speakers, and under which conditions does this speaker-level approach prove effective?

## 2 Methods

### 2.1 Datasets

We employed four datasets in this study. The CLAC dataset was used to define the reference intervals (RIs), while three additional datasets, (1) COMMITMENT, (2) Mental Wellbeing at Sea, and (3) audRespire, served as probes for comparison. These probe datasets were chosen for two main reasons: (i) they were collected under substantially different conditions, enabling the assessment of both controlled laboratory settings and everyday-life or crowdsourced scenarios—yet their included speech tasks were designed to be comparable across these different settings; and (ii) they capture a broader range of medical and health-related states beyond a binary distinction between healthy controls and a specific disorder, thereby allowing investigation of factors such as stress levels and respiratory symptoms. Unfortunately, due to ethics restraints, the probe datasets cannot be released publicly.

#### 2.1.1 CLAC

The Crowdsourced Language Assessment Corpus (CLAC) (13) comprises recordings from 1,807 self-reported healthy English speakers. The speakers perform multiple speech tasks, and we focus on the tasks that overlap most closely with the tasks also contained in our datasets: read speech and sustained phonation of the vowel /a:/. Besides having a presumed low prevalence of unhealthy subjects, CLAC offers the advantage of being larger than other publicly available speech corpora for the study of speech as a biomarker.

#### 2.1.2 COMMITMENT

The COMMITMENT (*Prediction of Non-motor Symptoms in Fully Ambulatory MS Patients Using Vocal Biomarkers*) dataset consists of 50 fully ambulatory people with multiple sclerosis (pwMS) (37 females) and 20 control participants (14 females). All participants were assessed for fatigue using the Fatigue Scale for Motor and Cognitive Functions (FSMC) (14). Speech samples in Swiss German were recorded in a quiet clinical setting at the Inselspital, an affiliate of the University of Bern, using the AI SoundLab^1^ framework, as introduced in Gonzalez-Machorro et al. (8) and Hammer et al. (9). Participants with MS exhibited low levels of disability, with a median Expanded Disability Status Scale (EDSS) score of 1.0—indicating minimal impairment—and a min/max EDSS score of 0.0/3.0, corresponding to no disability to moderate disability, while still being able to walk unaided (15). Fatigue within the pwMS was assessed as part of the COMMITMENT trial, and of the 50 pwMS, 17 female and 1 male participant were falling under the “clinical fatigue” category of the FSMC; due to that data sparsity, analysis was performed without regarding male pwMS in the fatigue/no fatigue condition.

Each voice recording session comprised of several speech tasks, as mentioned in Gonzalez-Machorro et al. (8). For this study, we only focus on sustained /a:/, corresponding to each patient sustaining the vowel “a” until reaching their maximum phonation time (MPT), and the text passage North Wind and the Sun (NWS) [Der Nordwind und die Sonne] (16), which was read by each speaker at the beginning and at the end of each session.

#### 2.1.3 Mental wellbeing at sea

The Mental Wellbeing at Sea dataset was introduced in Hecker et al. (10, 11), using the same voice recording framework, AI SoundLab, deployed on board of an oil tanker. In total, we collected data consisting of 378 survey sessions from 25 seafarers of 4 different nationalities over 9 weeks. Cohort’s demographics are presented in Hecket et al. (10). Each speaker recorded their data using their own device in this very confined environment, and therefore it consists of particularly noisy speech samples.

In each session, participants were asked to fill in questionnaires on their mental health and wellbeing along with speech tasks such as sustaining the vowel /a:/ until reaching their MPT and reading a language-independent sentence. That sentence was composed to cover many different vowels while keeping a simple syllable structure with consonant-vowel-chains (“Nilago me bu leffi, nulato dupo sam”), following the methodology described in Scherer et al. (17). The “symptomatic” category for this dataset was constructed by taking the continuous rating of “stress at recent work tasks” on a visual analogue scale (VAS) [0–100 range, see (10)] and binarising it as “low stress” for values smaller than 25 and “high stress” for values larger and equal of 25. This cut-off point resembled 80% of samples to lie within the “low stress” category and the remaining 20% of samples within the “high stress” category. Of the 25 participants, only 3 were female. Due to data sparsity, we omit these 3 female participants from the analyses.

#### 2.1.4 audRespire

The audRespire dataset is a crowdsourced longitudinal dataset introduced by Hecker et al. (12). It consists of individuals who participated in a data donation on respiratory symptoms. As the other datasets, it was collected using AI SoundLab. Data collection started on the 4th of January 2021, and the latest data point covered in this publication is from the 10th of September 2025. In total, 168 speakers have collected their speech data through 308 recorded sessions. After excluding speakers with self-reported voice or articulation disorders and those who did not disclose their sex, 161 speakers (54 female, 107 male) contributing 287 sessions (79 female, 208 male) remained for the sex-specific analyses.

In each session, participants were asked to complete a self-assessment questionnaire regarding their respiratory health at the time of recording. Self-reported predispositions of the speaker, such as whether they have voice or articulation disorders, were also recorded. We filtered out speakers who declared having speech impairments. Participants exhibiting respiratory symptoms were grouped under the “symptomatic” category. Furthermore, each participant was asked to sustain the vowel /a:/ until reaching MPT and to read the first two sentences of the NWS passage.

### 2.2 Reference interval framework

#### 2.2.1 Enhancing the reference interval framework through noise augmentation

The “Mental Wellbeing at Sea” and “audRespire” datasets were recorded on users’ smartphones under everyday-life conditions. Because noise can substantially alter feature values and some features are particularly noise-sensitive, we augmented CLAC—the corpus used to calculate the RIs bounds—with synthetic noise to emulate the recording conditions of these datasets. To quantify the noise profile of these datasets, we utilised the non-intrusive speech quality assessment (NISQA) v2.0 model to obtain mean opinion score (MOS) ratings for all samples within all datasets (18). The median MOS score from all samples of our everyday-life datasets was used as a reference to approximate its distribution. Noise was being added incrementally to CLAC: the MOS of batches of 500 samples was calculated, and in each batch, 70% of samples with a higher MOS were selected for augmentation. Of those 70% of files, 50% were augmented by mixing in samples labelled as “background noise” in the music, speech, and noise corpus (MUSAN) (19). Further 30% of the files-to-augment were mixed with white noise, and the remaining 20% were augmented by adding reverberation. After each pass, the median MOS of the batch and the one of the everyday life datasets were compared and if they still diverged by 0.2, a new round of noise augmentations was applied to the same batch with up to 5 iterations.

#### 2.2.2 Deriving RIs for interpretable acoustic features

We compute RIs for a set of 28 interpretable acoustic features, by defining the interval between the 2.5th and the 97.5th percentiles, with confidence intervals (CIs) on the upper and lower bounds, as described in Botelho et al. (3). The 28 features, previously used in Botelho et al. (3) and extracted with Praat (20) through the Python package praat-parselmouth (21), include dimensions associated with speech rhythm (e.g., speech rate, articulation rate, pause-related features), voice quality (e.g., jitter, shimmer, and F0-related features), and vocal tract shape (including format-related features). RIs were derived separately for different speech tasks (e.g., “read speech” and “sustained utterance /a:/”) and genders, following Botelho et al. (3). For the sustained /a:/, only voice quality and vocal tract shape features were extracted, resulting in a set of 20 features. We further compare the noise-augmented and the original versions of CLAC and calculated the RIs based on both datasets respectively. All three probe datasets were evaluated against both the clean and noise-augmented versions of CLAC RIs; results were identical after false discovery rate (FDR) correction (see Section 2.2.3.1).

#### 2.2.3 Using RIs to quantify different health states

After deriving RIs for interpretable acoustic features, we compare them to the acoustic features extracted from the probe datasets described in Section 2.1. Data to be evaluated using the RI approach were partitioned along the factors of gender, speech tasks, and dataset. For each partition, we normalised data based on the “control” population.^2^ Specifically, each feature was z-score normalised (zero-mean and unit-variance) using the mean and standard deviation of the control speakers within the same partition, following the normalisation approach of Botelho et al. (3): $z_{i}=(x_{i}-\mu_{\mathrm{ctrl}})/\sigma_{\mathrm{ctrl}}$. Per-partition normalisation parameters are provided in the Supplementary Data Sheet 1 and in Supplementary Presentation 1.pdf in Supplementary Section S1). To assess how our data were reflected in the framing of the RIs, we counted the number of samples that fell outside their bounds. In Botelho et al. (3), this was done sample-wise: for each audio sample, the number of features outside the RIs was computed. In contrast, here we focus on identifying which features are most informative for each target health state. Thus, our analysis is feature-wise: for each feature, we calculate the proportion of samples outside its RI. A feature is considered informative for a health state if a higher proportion of symptomatic samples than control samples fall outside its RI.

This metric, i.e., the proportion of samples outside the RI for each feature, was tested for significance using the Mann-Whitney *U* test, given that we could not assume normality. We concentrated on the proportion of features falling outside the RIs, which serves as a compact yet informative indicator of deviation from healthy acoustic patterns.

In addition to the binary inside/outside indicator, we systematically evaluated all five per-feature deviation scores proposed by Botelho et al. (3): ${\mathrm{DS}}_{\mathrm{RI}}$ (distance to the nearest RI bound, normalised by RI half-width), ${\mathrm{DS}}_{\mathrm{MSTD}}$ and its uncapped variant (deviation from the reference mean, normalised by standard deviation), ${\mathrm{DS}}_{Q123}$ (deviation from the reference interquartile range, normalised by interquartile range (IQR) half-width), and ${\mathrm{DS}}_{\mathrm{Mahalanobis}}$ (Mahalanobis distance incorporating the variance-covariance structure). ${\mathrm{DS}}_{\mathrm{Mahalanobis}}$ was excluded because robust covariance estimation for 28 features requires sample sizes larger than several of our analysis partitions. Based on feature selection using FDR correction (see Section 2.2.3.1), ${\mathrm{DS}}_{Q123}$ was selected as the primary RI-derived continuous metric. For each feature, the ${\mathrm{DS}}_{Q123}$ score is zero when a sample’s normalised value falls within the interquartile range (IQR; $Q_{1}$ to $Q_{3}$) of the healthy reference population, and otherwise equals the signed distance to the nearest IQR bound normalised by half the IQR width. Notably, ${\mathrm{DS}}_{Q123}$ uses the IQR rather than the explicit RI bounds (2.5th–97.5th percentiles), thereby defining a narrower healthy core that captures approximately 50% of the reference distribution, rather than the approximately 95% of the RI approach.

##### 2.2.3.1 Baseline comparison

To identify the acoustic features that most effectively distinguish “control” from “symptomatic” speakers, we compared our RIs-based approach to a conventional baseline. In that baseline, we directly tested the raw feature values for statistical significance between the two groups, without applying the RI framework. This comparison allows us to evaluate the added value of RIs—not only in terms of interpretability and robustness to noise, but also in highlighting deviations from normative acoustic patterns. By contrasting both methods, we aim to determine whether RIs can offer a more clinically meaningful and generalisable way to detect health-related changes in speech. Raw feature values were aligned with the symptomatic and control labels and directly tested with the Mann–Whitney *U* test. Cohen’s d “effect size” was computed to assess the overlap between the two groups. All population-level p-values from both approaches were corrected for multiple comparisons using the Benjamini–Hochberg (BH) false discovery rate (FDR) procedure at $p_{\mathrm{adj}}<0.05$, applied globally across all 288 valid tests^3^ within each approach.

#### 2.2.4 Individual-level assessment

To evaluate the potential of RIs for individual-level health assessment, we extended the population-level analysis (Section 2.2.3) with per-speaker deviation scoring. For every individual speech recording, we first normalised each acoustic feature using the partition-specific healthy speakers. For each feature, we checked whether its normalised value fell inside or outside the 95% healthy RI (2.5th–97.5th percentile) estimated on the CLAC corpus. From this, we derived two measures per recording: the *proportion of features outside the RI* (how many features are affected) and the *mean absolute distance from the nearest RI bound* for features that lie outside (how far they deviate). The composite *deviation score* combines both by multiplying the proportion of outside features with one plus the mean absolute distance, so that speakers who have many features far outside the healthy range receive a high score, whereas speakers whose features all remain inside the RI receive a score of zero.

Note that the per-feature deviation scores from Botelho et al. (3) used in the population-level analysis and the composite per-speaker deviation score serve different purposes: the former quantifies each feature’s deviation for group comparison, while the latter aggregates across all features to characterise each speaker’s overall acoustic profile relative to the healthy reference. In particular, ${\mathrm{DS}}_{Q123}$—which uses the IQR as the normative range—was not applied for individual profiling: a score anchored to the central 50% of the reference distribution assigns non-zero values to approximately half of healthy speakers by construction, undermining the clinical interpretability of the per-speaker profile. The standard RI bounds (2.5th–97.5th percentile), which capture 95% of the healthy population, provide the clinically-established threshold for individual assessment.

For the COMMITMENT dataset, where each speaker contributed a single recording session, the deviation score is computed directly from that session; for the read speech task, only the first NWS prompt was used, whereas the population-level analysis included both. For audRespire and MWaS, where labels may vary across sessions, the analysis was performed at the session level (one deviation score per speaker per session).

To quantify how well the deviation score discriminates between the symptomatic and control groups, we used the area under the curve (AUC) of the receiver operating characteristic (ROC) curve. The AUC equals the probability that a randomly drawn symptomatic speaker has a higher deviation score than a randomly drawn control speaker (22), making it a natural, threshold-free summary of discriminatory power for any continuous score. We report 95% bootstrap confidence intervals (1,000 iterations) with symptomatic speakers as the positive class. For partitions where exact enumeration of all label permutations is computationally feasible, we additionally report an exact permutation p-value as the proportion of permuted AUC values at least as large as the observed AUC.

## 3 Results

**Figure 1:**
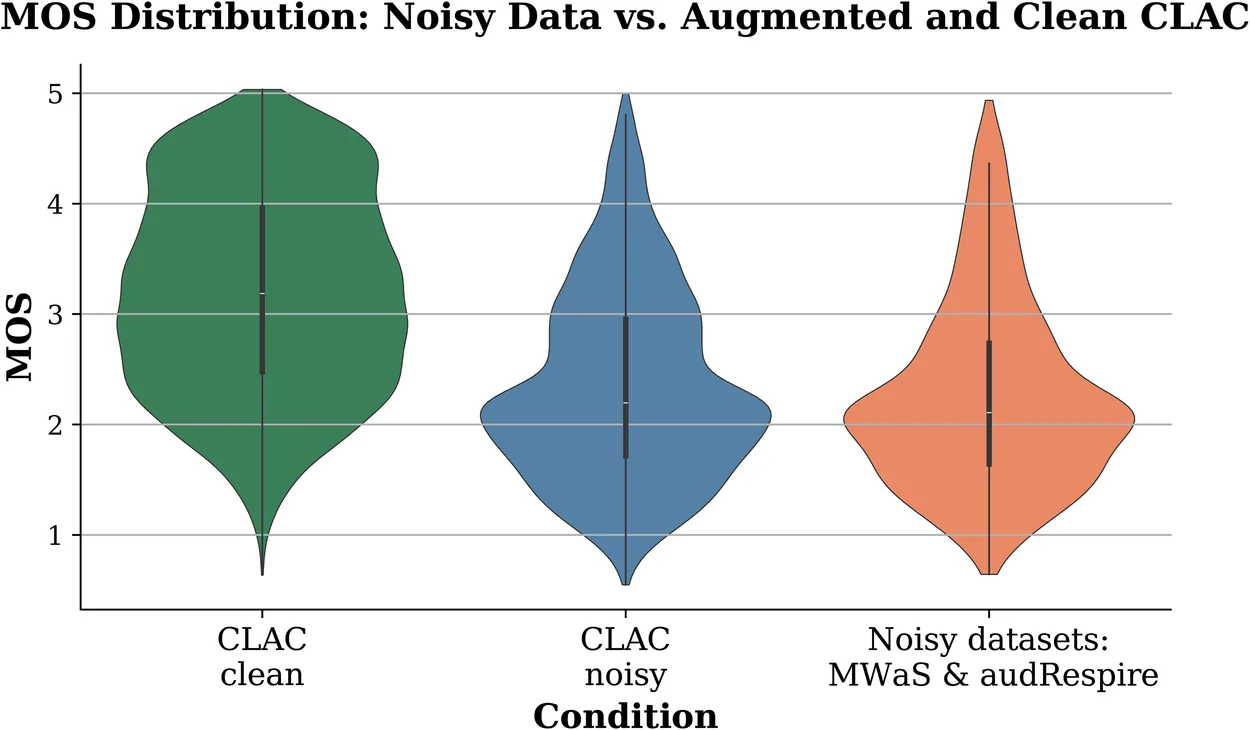
Mean opinion score (MOS) distribution (lower MOS indicates worse speech quality) of the non-augmented (“clean”) Crowdsourced Language Assessment Corpus (CLAC), the noise-augmented CLAC, and the pooled-together datasets under everyday-life conditions (Mental Wellbeing at Sea (MWaS) and audRespire).

The results of the noise augmentation of CLAC can be seen in Figure 1. It shows the MOS distribution of CLAC’s samples before (“clean”) and after (“noisy”) noise augmentation, as well as datasets collected under noisy, everyday-life conditions (“Mental Wellbeing at Sea” and “audRespire”). The shape of the MOS distribution of the noise-augmented CLAC approximates the one of the everyday-life datasets closely.

RIs based on the clean and noise-augmented version of CLAC were calculated. The feature-wise proportion of the number samples lying outside of the RIs was used as a metric to compare control and symptomatic populations.

All statistical comparisons are within-dataset; no cross-dataset statistical testing was performed. The conventional baseline, where feature values were tested directly for significance (see Section 2.2.3.1), and the RI approach, where the proportion of features outside of the RIs was assessed (see Section 2.2.3), are compared. All p-values were corrected for multiple comparisons using the Benjamini–Hochberg (BH) FDR procedure; Table 1 lists the baseline features that remained significant after FDR correction at $p_{\mathrm{adj}}<0.05$. The baseline statistical comparison yielded 23 of 288 valid, FDR-corrected significant features ($p_{\mathrm{adj}}<0.05$; 56 uncorrected). The RI-based approach, calculating the proportion of samples per feature outside of the RI yielded 0 of 288 valid, FDR-corrected significant features (20 uncorrected).

**Table 1:** Baseline features that remained significant after Benjamini–Hochberg (BH) false discovery rate (FDR) correction ($p_{\mathrm{adj}}<0.05$) across all 288 valid Mann–Whitney *U* tests.

Dataset	Sex	Task	Feature	Cohen’s d	p	$p_{\mathrm{adj}}$
audRespire	Female	/a:/	f1_median${}^{†}$	−0.91	<0.001	0.011
			f1_mean	−0.83	0.001	0.021
	Female	Read	silence_rate	0.57	0.003	0.038
	Male	/a:/	localShimmer${}^{†}$	−0.57	<0.001	0.004
			localdbShimmer${}^{†}$	−0.62	<0.001	0.004
			apq3Shimmer${}^{†}$	−0.52	<0.001	0.005
			aqpq5Shimmer${}^{†}$	−0.51	<0.001	0.006
			f2_mean${}^{†}$	−0.51	<0.001	0.011
			f2_median${}^{†}$	−0.49	<0.001	0.011
			apq11Shimmer	−0.28	0.001	0.021
			hnr	0.36	0.002	0.037
			localJitter	−0.24	0.004	0.050
			rapJitter	−0.23	0.004	0.050
	Male	Read	mean_speech_dur${}^{†}$	0.43	<0.001	0.009
			meanF0	−0.10	<0.001	0.010
			localabsoluteJitter${}^{†}$	0.31	<0.001	0.010
			mean_sil_count${}^{†}$	−0.51	0.001	0.021
MWaS	Male	/a:/	apq11Shimmer	−0.48	<0.001	0.012
			apq3Shimmer	−0.47	0.002	0.034
			localShimmer	−0.46	0.002	0.037
			aqpq5Shimmer	−0.37	0.002	0.037
			f1_mean	0.50	0.004	0.050
MS Binary	Male	Read	mean_pause_dur	−2.14	0.001	0.027

Population-level view on the data. Features are grouped by dataset, sex, and speech task. Cohen’s d indicates the effect size between control and symptomatic groups; computed as (symptomatic mean − control mean)/pooled standard deviation (SD), so negative values indicate a lower mean in the symptomatic group.

${}^{†}$Features that also remained significant after FDR correction under the ${\mathrm{DS}}_{Q123}$ continuous reference interval (RI) deviation score (3); two additional features also passed ${\mathrm{DS}}_{Q123}$ FDR correction in partitions not listed here: f1_median in MWaS male read speech ($p_{\mathrm{adj}}=0.047$) and meanF0 in MS fatigue female read speech ($p_{\mathrm{adj}}=0.043$).

No RI-based features remained significant after FDR correction, likely because binarising continuous values into inside/outside indicators reduces the Mann–Whitney *U* test to a proportions test, which has substantially less statistical power than testing continuous ranks. However, using the ${\mathrm{DS}}_{Q123}$ continuous deviation score (3), 12 of 288 features remained significant after global FDR correction ($p_{\mathrm{adj}}<0.05$); 10 of these are exact partition matches with the baseline (Table 1, marked with †), while the remaining two (MWaS male read speech f1_median and COMMITMENT female read speech meanF0) involve features that also appear in the baseline but in different partitions. Results are identical for both clean and noise-augmented CLAC reference intervals, confirming robustness to noise augmentation. Among the four tested metrics from Botelho et al. (3), ${\mathrm{DS}}_{Q123}$ retains the most features (12), followed by ${\mathrm{DS}}_{\mathrm{MSTD}}$ and its uncapped variant (10 each), while ${\mathrm{DS}}_{\mathrm{RI}}$ yields no significant features, likely due to zero inflation from collapsing all within-interval values to a score of zero, which generates excessive ties in the Mann–Whitney *U* test. The individual-level deviation score (Section 2.2.4) aggregates information across all features simultaneously, providing an additional perspective that does not require per-feature significance.

To assess the impact of the noise augmentation approach, we performed a Wilcoxon signed-rank test on the per-feature span (proportion outside the RI in the symptomatic group minus the control group) across all 288 valid feature-partition tests, pairing each feature under clean and noise-augmented CLAC RIs. The test was not significant (T=6645.5, p=0.055, matched-pairs rank-biserial r=−0.17, $n_{\mathrm{eff}}=178$ after excluding 110 tied pairs). Of the 178 features with differing spans, 107 favoured the clean and 71 the noise-augmented condition, indicating no evidence that noise augmentation improves population-level separation.

### 3.1 Individual-level assessment

Table 2 reports the per-speaker deviation scores and classification performance for each partition. Among all partitions, male pwMS performing the read speech task showed the strongest discrimination, with an AUC of 0.97[0.88,1.00], confirmed by exact permutation testing (p<0.001, BH-adjusted $p_{\mathrm{adj}}=0.001$; Supplementary Section S3). The corresponding male vowel /a:/ partition showed a similarly marked separation (AUC=0.94[0.78,1.00]). Given the small sample size (n=19), several robustness analyses were conducted to rule out artefacts due to feature redundancy, sex confounding, or chance: feature-family decorrelation, a combined-sex analysis with sex as covariate, leave-one-out stability, and a negative-control comparison using a non-MS dataset at matched sample size (Supplementary Sections S4–S7).

**Table 2:** Per-speaker deviation scores and classification performance.

Dataset	Sex	Task	$n_{S}$	$n_{C}$	AUC [95% CI]	${\bar{d}}_{S}$	${\bar{d}}_{C}$
MS Binary	Male	Read	13	6	0.97 [0.88, 1.00]	0.42	0.05
MS Binary	Male	/a:/	13	6	0.94 [0.78, 1.00]	0.70	0.16
MS Binary	Female	Read	37	14	0.61 [0.45, 0.77]	0.14	0.08
MS Binary	Female	/a:/	37	14	0.70 [0.54, 0.85]	0.46	0.16
MS Fatigue	Female	Read	17	20	0.66 [0.48, 0.83]	0.09	0.07
MS Fatigue	Female	/a:/	17	20	0.63 [0.45, 0.82]	0.19	0.13
audRespire	Male	Read	86	115	0.50 [0.41, 0.63]	0.12	0.10
audRespire	Male	/a:/	75	90	0.48 [0.35, 0.62]	0.15	0.14
audRespire	Female	Read	32	45	0.40 [0.31, 0.53]	0.03	0.09
audRespire	Female	/a:/	32	47	0.49 [0.36, 0.66]	0.09	0.09
MWaS	Male	Read	69	253	0.54 [0.43, 0.66]	0.13	0.09
MWaS	Male	/a:/	65	234	0.56 [0.42, 0.68]	0.29	0.20

Individual-level view on the data. The sample size (n) denotes the number of analysis units: speakers for MS (one recording session per speaker) and sessions for audRespire and MWaS (longitudinal designs where speakers may contribute multiple sessions). Area under the curve (AUC) with 95% bootstrap confidence interval (CI, 1, 000 iterations, symptomatic = “positive class”; speaker-clustered for audRespire and MWaS). $\bar{d}$: mean deviation score of the symptomatic (${\bar{d}}_{S}$) and control (${\bar{d}}_{C}$) participants.

Male multiple sclerosis (MS) fatigue and female Mental Wellbeing at Sea (MWaS) partitions omitted due to too small sample size.

Female MS participants showed moderate discrimination (read speech: AUC=0.61[0.45,0.77], /a:/: AUC=0.70[0.54,0.85]), with substantially smaller group differences in mean deviation scores. Whether this is attributable to genuine gender-related differences in acoustic manifestation, to the larger and more heterogeneous female cohort (n=51 vs. n=19 for males), or to a combination of both remains an open question. The MS fatigue sub-analysis yielded mixed results: the male partitions were excluded from Table 2 because the partition contains only 1 symptomatic speaker out of 13, rendering any reported metric an artefact of that single case. Female participants showed above-chance-level performance but modest discrimination (read speech: AUC=0.66[0.48,0.83], /a:/: AUC=0.63[0.45,0.82]), consistent with the weak population-level signal observed for the fatigue analysis.

In contrast, audRespire and MWaS showed near-chance-level classification performance (AUC 0.40–0.56), with mean deviation scores largely overlapping between control and symptomatic groups. This indicates that the individual-level RI assessment does not reliably distinguish symptomatic participants in these datasets.

**Figure 2:**
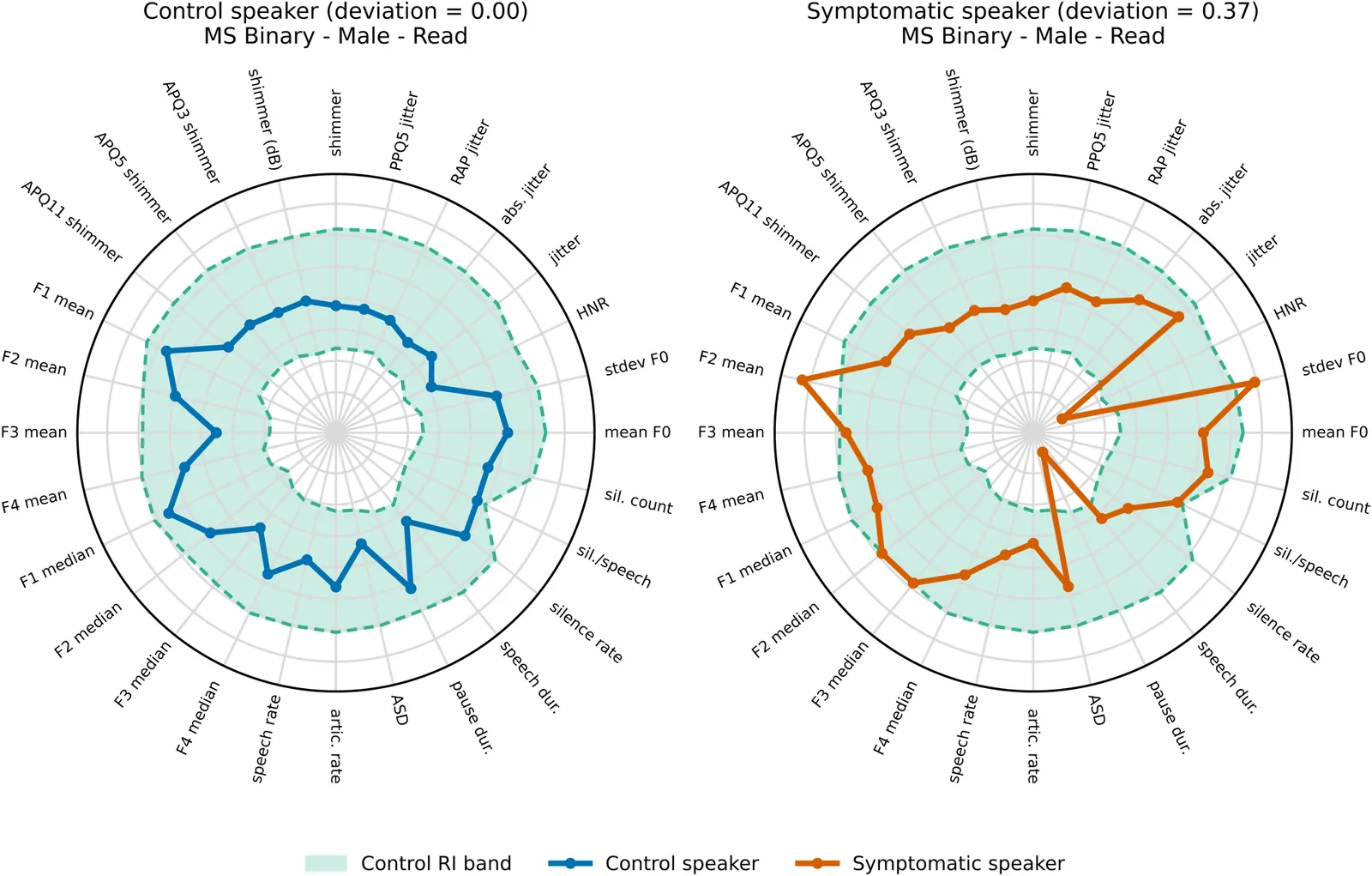
Radar plot profiles of one control (left) and one symptomatic (right) speaker from the male people with multiple sclerosis (pwMS) read speech partition of the COMMITMENT dataset. Speakers were selected as those closest to the median deviation score within their respective group. The reference interval (RI) bounds are outlined. The symptomatic speaker’s profile exceeds the RI for multiple features simultaneously.

Figure 2 shows the acoustic feature profile of a single control speaker and a single symptomatic speaker from the male pwMS read speech partition, with each feature normalised against the healthy reference population. To avoid selective presentation of extreme cases, speakers were selected as those closest to the median deviation score within their respective group. The symptomatic speaker’s profile exceeds the healthy RI across multiple features simultaneously, whereas the control speaker’s profile remains within the healthy bounds of the RIs.

## 4 Discussion

In this study, we evaluated the approach to use reference intervals (RIs) for acoustic features and applied it to three very distinct speech datasets. To account for the different levels of noise in these datasets, we augmented the data basis for the RIs, CLAC, with noise to make it more robust. To assess the RI-based approach, we calculated the proportion of samples outside of the RI bounds and tested for significance across the control and symptomatic populations—both for the noise-augmented and the clean data basis. To compare against an unadjusted approach, we tested raw feature values between the two populations without applying the RIs (i.e., baseline analysis). Results were heterogeneous. Noise augmentation of the reference corpus did not significantly alter population-level separation (Wilcoxon signed-rank test on per-feature spans, p=0.055). This is additionally underlined by identical FDR-corrected feature sets under ${\mathrm{DS}}_{Q123}$, which confirms that the RI framework is robust to the specific noise conditions introduced here. This indicates that moderate differences in reference corpus noise levels do not alter the analytical conclusions, which is relevant whenever the acoustic environment of the reference corpus cannot be closely matched to that of new probe recordings.

After FDR correction, the binary RI-based approach yields no significant features, while the baseline retains 23. This reflects the inherent power reduction from binarising continuous values into inside/outside indicators: the resulting proportions test has substantially less power than rank-based testing on continuous data. When the continuous ${\mathrm{DS}}_{Q123}$ deviation score from Botelho et al. (3) is used instead, 12 features remained significant after FDR correction; 10 are exact partition matches with the baseline, suggesting that the RI framework captures largely the same underlying signal as the baseline, albeit with reduced sensitivity.

The success of ${\mathrm{DS}}_{Q123}$—despite not using the explicit RI bounds—highlights an important methodological point. ${\mathrm{DS}}_{Q123}$ uses the IQR ($Q_{1}$–$Q_{3}$), which captures approximately 50% of the reference distribution, compared to the RI’s approximately 95% (2.5th–97.5th percentiles). The narrower normative range ensures that more samples receive non-zero deviation scores, preserving Mann–Whitney *U* test rank information that the binary RI indicator negates through excessive ties. This suggests that the RI framework’s value for population-level group comparison lies not in the specific percentile thresholds but in the general principle of characterising a healthy reference population and measuring deviation from it. The explicit RI bounds remain valuable for interpretability—classifying individual features as “within” or “outside” normal limits—and for the individual-level composite score, where the 95% interval provides a clinically meaningful boundary.

The features that remain significant after this baseline FDR filter are concentrated in voice quality (shimmer, jitter) and prosodic features across audRespire and MWaS, with a single MS feature (mean pause duration) exhibiting the largest effect size (d=−2.14). We examine the baseline features that remain significant after FDR correction (Table 1) and put these findings into context with literature.

Regarding the audRespire dataset in particular, the majority of features that remained significant after FDR correction (17 of 23) were voice quality features (shimmer, jitter, HNR) in male speakers, while formant features dominated in female speakers. This is in line with literature, in particular COVID-19, where these voice quality features are especially affected (23).

Speakers in the MWaS dataset were disproportionately exposed to work-related stress. Shimmer-related features^4^ emerged, which aligns with previous research on speech-based stress detection (24). That research demonstrated that shimmer features, which quantify amplitude perturbations in voice signals, are particularly sensitive to stress and psychological arousal.

For MS, only mean pause duration in male read speech remains significant after FDR correction, with the largest effect size in the table (Cohen’s d=−2.14, indicating shorter mean pause durations in pwMS relative to controls). Although the MS speech literature predominantly reports longer and more frequent pauses (25, 26), the reversed direction observed here may reflect the low clinical severity of this cohort (median EDSS=1.0) or task-specific pacing behaviour during read speech. The prominent effect size is consistent with the high individual-level discrimination observed for this partition; leave-one-out analysis confirms that no single speaker drives the effect, and the 95% bootstrap CI excludes zero (Supplementary Section S2).

At the individual level, the deviation score adds a complementary perspective. For male pwMS performing read speech, a single composite score achieves promising discrimination from controls (AUC=0.97 for read speech and 0.94 for /a:/), supported by exact permutation testing and multiple robustness analyses (Supplementary Sections S3–S8).

As a single-feature baseline, per-feature AUC values using |z-score| as classifier were computed: the best individual feature (mean pause duration, AUC=0.87) falls short of the composite, confirming that multi-feature aggregation captures complementary information (Supplementary Section S8). These results are nonetheless based on partitions of only 19 speakers and should be interpreted as a promising preliminary signal that warrants replication in larger cohorts. A combined-sex analysis (n=70) confirms that the deviation score is a significant predictor of MS status after controlling for sex (logistic regression, p<0.02), and sex itself is not a significant independent predictor (p>0.12; Supplementary Section S6).

Female pwMS show more moderate separation, possibly reflecting larger cohort heterogeneity. Given the low clinical severity of the COMMITMENT cohort (median EDSS = 1.0, all participants fully ambulatory), the observed separation is noteworthy: the per-speaker score preserves individual deviations that group-level tests tend to dilute. The successful transfer of RIs estimated on English-speaking controls to Swiss German speakers is consistent with observations in Gonzalez-Machorro et al. (15). For audRespire and MWaS, the individual-level deviation scores show limited discriminative power (AUC 0.40–0.56).

The complementary strengths of the two levels of analysis are particularly evident when comparing audRespire and COMMITMENT. At the population level, audRespire contributes 17 of 23 baseline features that remain significant after FDR correction, reflecting diffuse but statistically detectable per-feature effects in a large cohort. At the individual level, however, these per-feature effects do not aggregate into a coherent per-speaker pattern: deviation scores remain near chance level. Conversely, for MS, only a single feature (mean pause duration) remains significant after population-level FDR correction, yet the composite deviation score achieves an AUC of 0.97.

This is analogous to the well-known ecological inference problem (27): group-level associations (many features differ on average) do not necessarily translate to individual-level patterns (speakers differ coherently across features), and vice versa. Comparably, acoustic features in cognitive assessment show numerous significant group-level associations with neuropsychological test scores, while their combination into composite scores yields only a modest incremental gain in individual-level mild cognitive impairment (MCI) prediction beyond demographic baseline predictors (28). The RI framework supports both perspectives from the same reference data, and their divergence is informative: it diagnoses whether a condition’s effects are diffuse across features or coherent within speakers.

## 5 Conclusion

We assess the potential of reference intervals (RIs) as a disorder-agnostic framework for speech-based health assessment, applying them at both the population and the individual level across three datasets targeting distinct health conditions. The two levels of analysis provide complementary perspectives: population-level testing identifies which features deviate from healthy norms across groups, proving most informative for respiratory conditions in larger cohorts; individual-level scoring identifies which speakers deviate across features simultaneously, proving most informative for neuromotor impairments. Noise augmentation of the healthy reference corpus yields similar population-level separation to clean reference intervals, demonstrating robustness of the RI framework to moderate noise augmentation. The continuous ${\mathrm{DS}}_{Q123}$ metric bridges the gap between statistical power and RI-grounded interpretability: unlike raw baseline statistical testing, it anchors each feature’s deviation to the healthy reference distribution, and unlike the binary RI indicator, it preserves the rank information needed for powerful non-parametric testing. This yields 12 globally FDR-corrected features, while retaining the per-feature clinical grounding of the RI framework. The per-speaker deviation score provides a complementary, interpretable indicator that captures individual acoustic deviations from the healthy reference—achieving high discrimination for MS, particularly in male read speech, despite the cohort’s low clinical severity.

For researchers applying the RI framework to a new speech dataset, both levels of analysis are recommended as a default workflow, since the same underlying reference data can be used. Whether population-level or individual-level analysis will be more informative depends on the nature of the condition: population-level testing is most powerful when a condition produces diffuse per-feature effects detectable across a large cohort (as seen for audRespire); individual-level scoring is most powerful when a condition causes correlated multi-feature deviations within speakers (as seen for MS).

Future work should focus on obtaining larger populations of healthy speech, so that more dataset-dependent variance could be reflected. Further, correlating the individual-level deviation score with other clinical severity scales to establish its utility for monitoring disease progression is another promising avenue. We present an approach that adapts the underlying healthy reference population to the noise characteristics of our probe datasets; refining this approach to better approximate naturalistic noise is a promising next step.

## Data Availability

The CLAC dataset is publicly available (https://sls.csail.mit.edu/downloads/clac/). Due to ethics constraints, the COMMITMENT, Mental Wellbeing at Sea, and audRespire datasets cannot be shared. Requests to access these datasets should be directed to Pascal.Hecker@hpi.de.

## Footnotes

^1^aisoundlab.audeering.com, powered by audEERING GmbH.

^2^We define controls as individuals without the target health state (e.g., when analysing fatigue in pwMS, “non-fatigued” subjects serve as controls).

^3^Each test corresponds to one feature in one analysis partition (dataset × sex × task). With 48 features per partition (28 read-speech + 20 vowel), seven partitions (2 audRespire + 1 MWaS + 2 MS-binary + 2 MS-fatigue) yield 336 total tests; excluding 48 male MS-fatigue tests (only 1 symptomatic speaker, insufficient for the Mann–Whitney *U* test) gives 288 valid tests.

^4^apq3Shimmer, aqpq5Shimmer, apq11Shimmer, localShimmer.

## Supplementary material

Supplementary Presentation 1 Supplementary sections S1–S8 with a detailed overview of the study pipeline, a population-level effect size investigation, and robustness analyses of the individual-level deviation score results.

Supplementary Data Sheet 1 Normalisation parameters of each partition for all probe datasets of this study.
