# Supplementary material for: Outside the healthy bounds: reference intervals for population and individual-level medical speech analysis
Source: Front Digit Health. 2026 Aug 19;8:1870061. doi: 10.3389/fdgth.2026.1870061 (PMC13535962; doi:10.3389/fdgth.2026.1870061)
Supplement: Supplementary file 1 [file Presentation1.pdf]

Supplementary Material

This Supplementary Material provides an overview of the study pipeline, a population-level effect size investigation, and detailed robustness analyses of the individual-level deviation score results reported in the main manuscript. Section S1 presents the analysis pipeline and normalisation parameters. Section S2 investigates the robustness of the mean pause duration effect size at the population level. Sections S3–S8 address the robustness of the individual-level analysis: permutation testing with multiple testing corrections (S3), individual data-point visualisation (S4), feature-family decorrelation (S5), combined-sex analysis with sex as covariate (S6), cohort robustness analyses (S7), and single-feature area under the curve (AUC) baseline comparison (S8). Individual-level robustness analyses (S3–S8) were performed on the COMMITMENT dataset (multiple sclerosis (MS) binary: people with multiple sclerosis (pwMS) vs. controls).

1 S1: STUDY PIPELINE

Figure S1 presents an overview of the analysis pipeline. The reference corpus (Crowdsourced Language Assessment Corpus (CLAC); 1,807 healthy speakers) provides the basis for reference interval (RI) calculation. Three probe datasets – COMMITMENT (MS), audRespire (respiratory symptoms), and Mental Wellbeing at Sea (MWaS) (occupational stress) – are partitioned by dataset, sex, and speech task, and z-score normalised using the control speakers within each partition. The normalised features are then evaluated against the RI bounds at two complementary levels: (1) population-level analysis tests per-feature deviation scores for group differences using the Mann–Whitney U test with Benjamini–Hochberg (BH) false discovery rate (FDR) correction; (2) individual-level analysis computes a per-speaker composite deviation score that quantifies each speaker's acoustic deviation from the healthy reference, and evaluates discriminative ability via receiver operating characteristic (ROC) area under the curve (AUC).

1.1 Normalisation Parameters

For each analysis partition (dataset $\times$ sex $\times$ task), features were z-score normalised (zero-mean and unit-variance normalisation) by fitting a "standard scaler" on the control group within that partition, following the normalisation and partitioning approach established by Botelho et al. (2024): $z_i = (x_i - \mu_{\text{ctrl}})/\sigma_{\text{ctrl}}$, where μ_{ctrl} and σ_{ctrl} are the mean and standard deviation of the control speakers for that feature in that partition. The normalisation parameters are specific to each partition and are provided as "Supplementary Data Sheet 1" (comma-separated values (CSV) format; 14 partitions $\times$ up to 28 features per partition). This ensures that population-level and individual-level analyses are comparable across partitions, while accounting for partition-specific distributional differences in the control population.

2 S2: EFFECT SIZE INVESTIGATION FOR MEAN PAUSE DURATION

The largest effect size in Table 1 of the main publication is Cohen's $d = -2.14$ for mean pause duration in the MS binary male read-speech partition ($n = 19$; 13 pwMS + 6 controls). Since $d =$ (symptomatic mean $-$ control mean)$/$pooled SD, the negative sign indicates that pwMS have *shorter* mean pause durations than controls. This contradicts the MS speech literature, which predominantly reports longer and more frequent pauses (Plotas et al., 2023; Kieling et al., 2023). We therefore investigate the robustness of this finding.

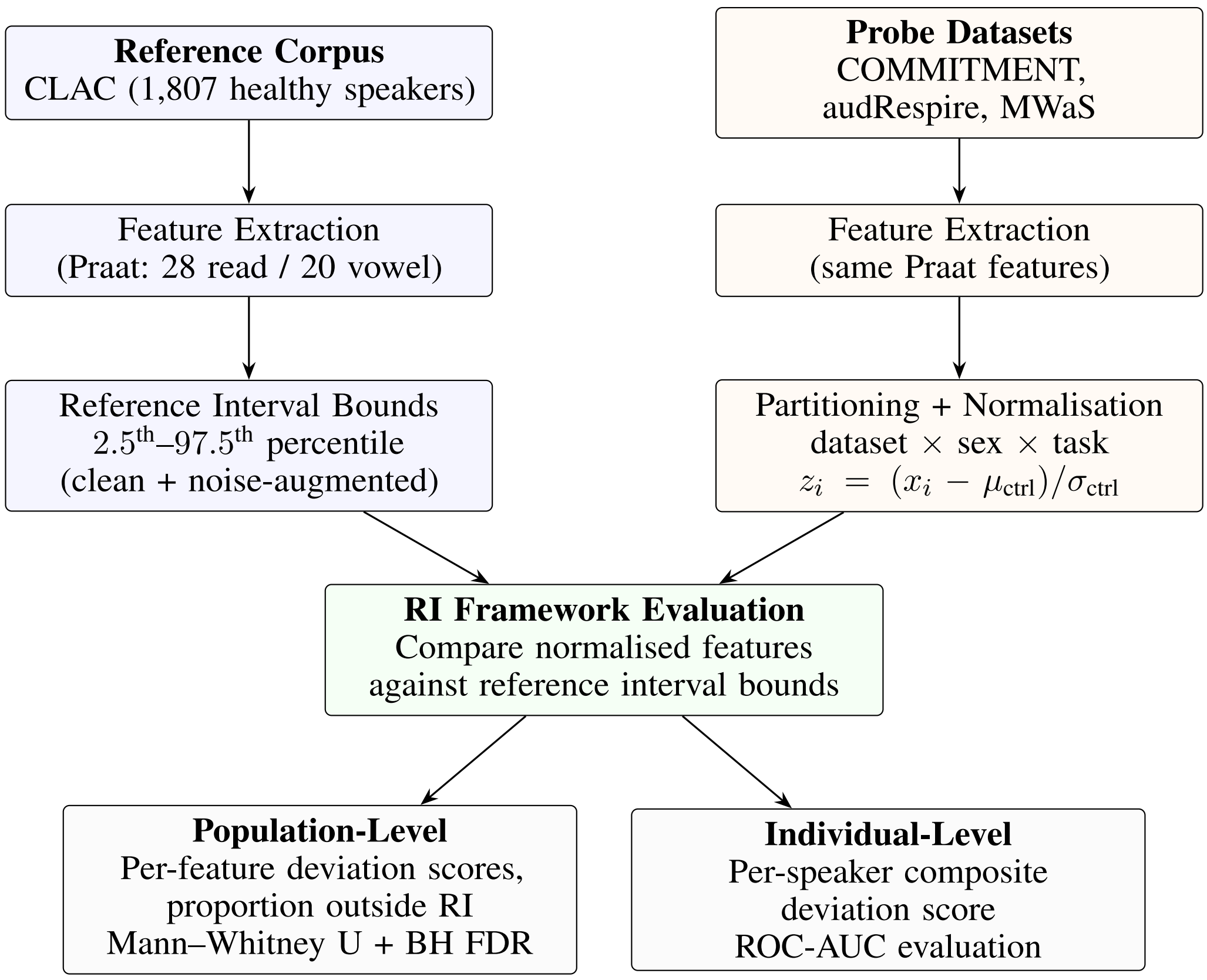

Figure S1. Overview of the analysis pipeline. The reference corpus (CLAC) provides healthy reference interval bounds; probe datasets are partitioned and z-score normalised using control speakers within each partition. Both components feed into population-level (per-feature group comparison) and individual-level (per-speaker deviation score) analyses.

2.1 Group-Level Statistics

Table S1 reports descriptive statistics for mean pause duration and other timing features. The control group ($n = 6$) has a higher mean pause duration (0.604 ± 0.060 s) than the pwMS group ($n = 13$, 0.484 ± 0.055 s). The shorter-pause pattern is specific to mean pause duration; other timing features show inconsistent or weak differences.

Table S1. Group summary for timing features in the male multiple sclerosis (MS) binary read-speech partition. pwMS = people with MS. ASD = average syllable duration (speaking time / number of syllables).

Feature	Control ($n = 6$)		pwMS ($n = 13$)	
	Mean	SD	Mean	SD
mean_pause_dur	0.604	0.060	0.484	0.055
mean_speech_dur	2.820	1.019	2.598	0.676
silence_rate	0.177	0.054	0.155	0.044
silence_speech_ratio	0.920	0.033	0.931	0.017
speechrate	4.228	0.541	4.017	0.368
articulation_rate	5.125	0.417	4.757	0.376
ASD	0.196	0.016	0.211	0.017
mean_sil_count	0.290	0.072	0.321	0.079

2.2 Leave-One-Out Cohen's *d* Stability

Each of the 19 speakers was removed in turn and Cohen's d was recomputed. All 19 leave-one-out d values remain negative (range: $[-2.73, -1.94]$, SD $= 0.20$), confirming that no single speaker drives the effect. The most influential speaker is a control participant whose removal shifts d by 0.59; removing this speaker yields $d = -2.73$ (stronger effect), indicating that this speaker partially attenuates the group difference.

2.3 Bootstrap Confidence Interval

A nonparametric bootstrap ($10{,}000$ iterations) yields a 95 % CI of $[-4.51, -1.10]$ for Cohen's d. The CI excludes zero, confirming that the effect is statistically significant despite the small sample.

2.4 Female Partition Comparison

In the female MS binary read-speech partition ($n = 51$; 37 pwMS + 14 controls), the Cohen's d for mean pause duration is -0.054, indicating essentially no difference. The shorter-pause pattern observed in males is therefore sex-specific and does not generalise to the female partition.

2.5 Summary

The $d = -2.14$ effect for mean pause duration is robust: no single speaker drives the effect (all leave-one-out (LOO) values negative), and the 95 % bootstrap CI excludes zero ($[-4.51, -1.10]$). However, the reversed direction relative to literature (shorter rather than longer pauses) may reflect the cohort's low clinical severity (median Expanded Disability Status Scale (EDSS) $= 1.0$), task-specific pacing constraints in read speech, or compensatory strategies in early-stage MS. The effect is sex-specific (female $d = -0.05$), consistent with the broader sex asymmetry observed throughout the analysis.

3 S3: PERMUTATION TEST RESULTS

The following sections (S3–S8) address the robustness of the individual-level deviation score analysis reported in the main manuscript.

To assess the statistical significance of the observed AUC values, permutation tests were conducted for all four MS binary partitions (2 sexes $\times$ 2 speech tasks). For the male partition ($n = 19$; 13 pwMS + 6 controls), all $\binom{19}{13} = 27{,}132$ possible label assignments were enumerated exhaustively (exact mode). For the female partition ($n = 51$; 37 pwMS + 14 controls), exact enumeration of $\binom{51}{14} \approx 1.29 \times 10^{12}$ permutations is computationally infeasible; instead, $100{,}000$ Monte Carlo iterations were used. The p-value is computed as the proportion of permuted AUC values at least as large as the observed AUC. The $27{,}132$ permutations in the exact mode construct a single null distribution; they are not separate hypothesis tests and do not require multiple testing correction.

However, the four permutation tests across partitions constitute a testing family. Table S2 reports Benjamini–Hochberg (BH) p_{adj}-values (FDR control) to correct for the four-test family. Both male partitions remain highly significant after correction. The female vowel partition ($p = 0.027$) remains significant after correction ($p_{adj} = 0.036$).

Table S2. Permutation test results for the four multiple sclerosis (MS) binary partitions, with multiple testing corrections. "Exact" indicates exhaustive enumeration of all label permutations; "MC" indicates Monte Carlo estimation with $100{,}000$ iterations. AUC = area under the receiver operating characteristic curve.

Sex	Task	AUC	p-value	BH p_{adj}	Mode
Male	Read speech	0.974	$<$0.001	0.001	Exact
Male	Vowel /a:/	0.936	0.001	0.003	Exact
Female	Vowel /a:/	0.701	0.027	0.036	MC
Female	Read speech	0.614	0.212	0.212	MC

4 S4: INDIVIDUAL DATA-POINT VISUALISATION

Figure S2 displays the deviation scores of all individual speakers across the four MS binary partitions, presented as strip plots overlaid on box plots. Each data point represents one speaker. The visual separation between pwMS and controls is most pronounced for the male partitions, consistent with the AUC values reported in Table 2 of the main manuscript. For the female partitions, the distributions overlap more substantially, reflecting the moderate AUC values.

5 S5: FEATURE-FAMILY DECORRELATION ANALYSIS

Multiple shimmer variants (apq3Shimmer, apq5Shimmer, localShimmer, localdbShimmer) and other correlated features may inflate the count of features outside the reference interval (RI), potentially biasing the deviation score. To assess the impact of feature redundancy, the 28 acoustic features (read speech) and 20 features (vowel) were grouped into seven feature families: fundamental frequency (F0), jitter, shimmer, harmonics-to-noise ratio (HNR), formants, rhythm, and pause. The *family-mean* aggregation strategy averages the normalised values within each family, yielding seven (read) or five (vowel) family-level features from which the deviation score is recomputed. This approach is objective and eliminates within-family redundancy entirely.

Table S3 reports the results. For male read speech, the AUC decreases from 0.974 (28 features) to 0.846 (7 family means), but remains significant (exact permutation $p = 0.014$). For male vowel, the AUC decreases from 0.936 to 0.859 ($p = 0.011$). These results indicate that the discrimination is not an artefact of correlated features: the signal persists even after aggressive decorrelation. As expected, the female partitions – which had weaker baseline signals – show reduced AUC values that do not reach significance after decorrelation. Negative-control datasets (audRespire, MWaS) yield AUC values near chance (0.47–0.56) under all strategies, confirming that feature redundancy does not systematically inflate AUC.

6 S6: COMBINED-SEX ANALYSIS WITH SEX AS COVARIATE

To clarify whether the high AUC observed in male participants reflects a genuine sex-specific effect or is attributable to the small sample size, male and female MS speakers were pooled into a single analysis ($n = 70$; 50 pwMS + 20 controls) for each speech task. Per-speaker deviation scores were computed using sex-specific RI bounds (unchanged from the per-sex analyses in the main manuscript). The pooled deviation scores were evaluated with (i) a stratified permutation test (permuting labels within sex strata, $100{,}000$ Monte Carlo iterations) and (ii) logistic regression with sex as a covariate.

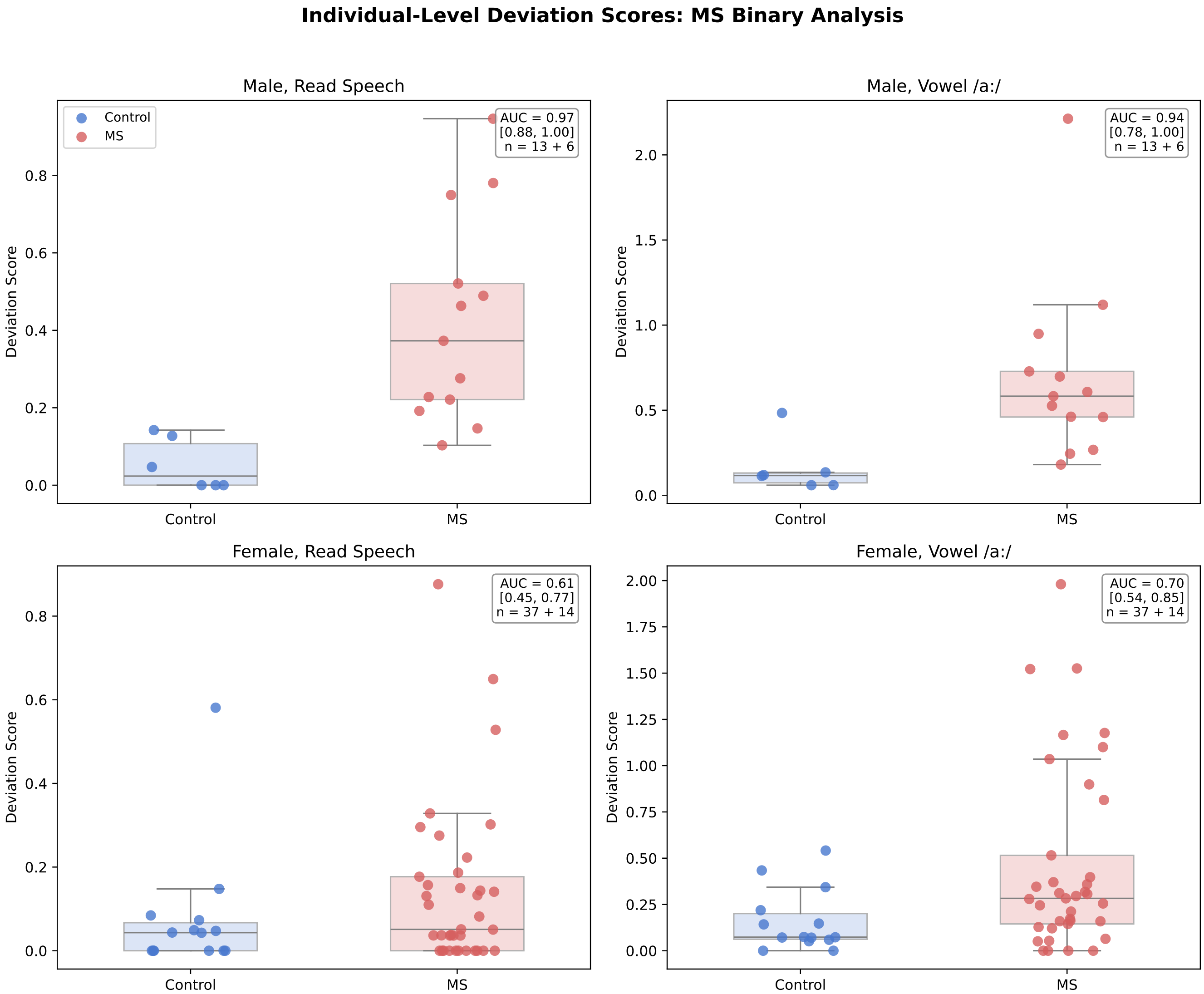

Figure S2. Individual deviation scores for all speakers in the four multiple sclerosis (MS) binary partitions (male/female $\times$ read speech/vowel /a:/). Each dot represents one speaker's composite deviation score. Box plots show the median, interquartile range, and whiskers. "disordered" = people with MS (pwMS); "healthy" = controls.

Table S3. Feature-family decorrelation results. "Full" uses all available features; "Family mean" uses one averaged value per feature family. p-values from exact permutation tests (male) or Monte Carlo permutation tests with $100{,}000$ iterations (female).

Partition	Strategy	N features	AUC	Perm. p
Male read	Full	28	0.974	<0.001
Male read	Family mean	7	0.846	0.014
Male vowel	Full	20	0.936	0.001
Male vowel	Family mean	5	0.859	0.011
Female read	Full	28	0.614	0.212
Female read	Family mean	7	0.529	0.615
Female vowel	Full	20	0.701	0.027
Female vowel	Family mean	5	0.606	0.175

Table S4 reports the pooled AUC and stratified permutation p-values. Both speech tasks yield significant discrimination after BH correction for two tests.

Table S4. Combined-sex analysis: pooled area under the curve (AUC) with stratified permutation test. p-values from Monte Carlo permutation with $100{,}000$ iterations, permuting within sex strata. Benjamini–Hochberg (BH) false discovery rate (FDR) correction applied for two tests.

Task	N (pos/neg)	AUC [95 % CI]	Strat. perm. p	BH p_{adj}
Read speech	50 / 20	0.71 [0.58, 0.83]	0.004	0.004
Vowel /a:/	50 / 20	0.77 [0.65, 0.88]	<0.001	<0.001

Table S5 reports the logistic regression results. The deviation score is a significant predictor of MS status ($p = 0.019$ for read speech, $p = 0.006$ for vowel) after controlling for sex. Crucially, sex itself is not a significant independent predictor ($p = 0.123$ for read, $p = 0.193$ for vowel), indicating that the high male AUC is not simply a sex artefact. The sex × deviation_score interaction term was tested in a separate model and found to be non-significant ($p = 0.137$ for read, $p = 0.194$ for vowel), suggesting that the deviation score operates in the same direction across sexes, with the male partition exhibiting a stronger effect.

Table S5. Logistic regression (label ∼ deviation_score + sex) for the combined-sex multiple sclerosis (MS) binary analysis. Positive coefficients indicate higher odds of MS status.

Task	Coeff. (dev. score)	p (dev. score)	Coeff. (sex=male)	p (sex)
Read speech	6.65	0.019	−1.11	0.123
Vowel /a:/	4.93	0.006	−0.94	0.193

7 S7: COHORT ROBUSTNESS

Given the small male MS partition ($n = 19$; 13 pwMS + 6 controls), several analyses were conducted to assess whether the observed AUC could be a “lucky” result driven by a single influential speaker or by the particular sample composition.

7.1 Leave-One-Out AUC Stability

Each of the 19 male speakers was removed in turn, and the AUC was recomputed on the remaining 18 speakers (Table S6). The LOO distribution comprises exactly $n = 19$ values – one per speaker – and the reported min/max range represents the complete LOO AUC distribution. No single speaker removal

Table S6. leave-one-out (LOO) area under the curve (AUC) stability for the male multiple sclerosis (MS) partition. Each value in the LOO distribution corresponds to the AUC obtained after removing one of the 19 speakers.

Metric	Read speech	Vowel /a:/
Full AUC	0.974	0.936
LOO min AUC	0.969	0.923
LOO max AUC	1.000	1.000
LOO std AUC	0.007	0.017

changes the AUC by more than 0.005 (read speech) or 0.013 (vowel), indicating that the result is not driven by any individual speaker.

7.2 Sex Asymmetry and Effect Sizes

Table S7 compares AUC values, bootstrap confidence intervals, and effect sizes between male and female partitions. The male effect sizes (Cohen's $d = 1.62$ for read speech) substantially exceed the female values ($d = 0.35$), consistent with a genuine sex-related difference in acoustic manifestation rather than a small-sample artefact. The male control group ($n = 6$) is small, which widens the confidence intervals but does not invalidate the large effect size.

Table S7. Sex asymmetry in AUC and effect sizes for the multiple sclerosis (MS) binary analysis. AUC $95\,\%$ confidence intervals (CIs) from 500 bootstrap iterations.

Task	Male AUC	Male 95 % CI	Female AUC	Female 95 % CI	d male	d female
Read speech	0.974	[0.90, 1.00]	0.614	[0.44, 0.78]	1.62	0.35
Vowel /a:/	0.936	[0.79, 1.00]	0.701	[0.55, 0.86]	1.18	0.68

7.3 Bootstrap Probability of All-Nonzero Scores

All 13 male pwMS speakers have a non-zero deviation score (i.e., at least one feature outside the RI), whereas a proportion of female pwMS speakers have a deviation score of exactly zero. To assess how unlikely this pattern is, we computed the probability of observing all 13 male speakers with non-zero scores under the null hypothesis that each speaker independently has the same non-zero rate as the female group. For read speech, the female non-zero rate is 70.3 % (26/37), yielding an analytical probability of $0.703^{13} = 0.010$ and a bootstrap estimate of 0.012 (95 % CI: [0.010, 0.014]; $10{,}000$ iterations, Wilson binomial proportion CI). This indicates that the male pattern is statistically rare under the female base rate. For vowel, the female non-zero rate is higher (89.2 %, 47/51), yielding a probability of 0.23, suggesting the male advantage is less pronounced for this task.

7.4 Negative-Control Comparison (audRespire Dataset)

To test whether small sample size alone could produce inflated AUC values, the audRespire dataset (male participants) was used as a negative control. audRespire deviation scores were subsampled 500 times to match the MS cohort composition (13 positive + 6 negative speakers), and the AUC was computed for each subsample. The mean matched-sample AUC was 0.49 ($\pm$ 0.11) for read speech and 0.47 ($\pm$ 0.14) for vowel – both near chance level (0.50). This confirms that small sample size alone does not inflate AUC; the high MS AUC reflects a signal in the data.

7.5 Summary

The combination of exact permutation testing ($p < 0.001$), LOO stability (minimum AUC = 0.969), feature-family decorrelation (family-mean AUC = 0.846, $p = 0.014$), combined-sex analysis (deviation score significant after controlling for sex), and negative-control comparison (matched-sample AUC ≈ 0.49) indicates that the observed discrimination in the male MS partition is unlikely to be an artefact of small sample size, feature redundancy, or sex confounding. These results should nonetheless be interpreted as a preliminary signal that warrants replication on independent, larger cohorts.

8 S8: SINGLE-FEATURE AUC BASELINE

To assess whether the composite deviation score adds value beyond any single acoustic feature, ROC-AUC was computed for each feature individually, using the absolute z-score $|z|$ as the classifier score. Since features are z-score normalised against healthy controls, $|z|$ captures bidirectional deviations: both abnormally high and abnormally low feature values contribute to higher scores. This is consistent with the composite deviation score, which aggregates absolute distances from RI bounds. Single-feature AUC values were computed at the same aggregation level as the composite (speaker-level for MS, session-level for audRespire and MWaS), with speaker-clustered bootstrap 95% confidence intervals where applicable.

Table S8 reports the results for the four MS binary partitions. For the key partition (male read speech), the composite deviation score (AUC = 0.974) exceeds the best single feature (mean pause duration, AUC = 0.872) by ΔAUC = 0.103. Only two of 28 features achieve AUC > 0.8 individually. For male vowel, the composite (AUC = 0.936) marginally exceeds the best single feature (local absolute jitter, AUC = 0.923), with four features above AUC > 0.8. For the female partitions, where the composite itself shows moderate discrimination, single-feature AUCs are comparable. For audRespire and MWaS, both composite and single-feature AUCs cluster near chance (0.40–0.64), consistent with the limited individual-level discriminative power reported in the main text.

Table S8. Single-feature area under the curve (AUC) baseline for multiple sclerosis (MS) binary partitions. The top-3 features per partition are shown alongside the composite deviation score AUC. ΔAUC = composite $-$ single-feature AUC.

Partition	Feature	AUC	95% CI	ΔAUC
Male read speech (composite AUC = 0.974 [0.88, 1.00])				
	mean_pause_dur	0.872	[0.67, 1.00]	+0.103
	meanF0	0.833	[0.62, 1.00]	+0.141
	f2_median	0.769	[0.51, 0.96]	+0.205
Male vowel /a:/ (composite AUC = 0.936 [0.78, 1.00])				
	localabsoluteJitter	0.923	[0.79, 1.00]	+0.013
	hnr	0.872	[0.68, 1.00]	+0.064
	localJitter	0.859	[0.66, 1.00]	+0.077
Female read speech (composite AUC = 0.614 [0.45, 0.77])				
	f1_median	0.670	[0.50, 0.84]	−0.056
	speechrate	0.666	[0.48, 0.83]	−0.052
	articulation rate	0.662	[0.47, 0.83]	−0.048
Female vowel /a:/ (composite AUC = 0.701 [0.54, 0.85])				
	f4_mean	0.712	[0.57, 0.84]	−0.012
	f3_mean	0.712	[0.56, 0.84]	−0.012
	f4_median	0.685	[0.52, 0.82]	+0.016

The composite score's advantage is most pronounced in the male read-speech partition, where the multi-feature aggregation captures correlated deviations across prosodic, voice quality, and timing features that no single feature alone achieves.

REFERENCES

Botelho, C., Abad, A., Schultz, T., and Trancoso, I. (2024). Speech as a biomarker for disease detection. *IEEE Access*

Kieling, M. L. M., Finkelsztejn, A., Konzen, V. R., dos Santos, V. B., Ayres, A., Klein, I., et al. (2023). Articulatory speech measures can be related to the severity of multiple sclerosis. *Frontiers in Neurology* Volume 14 - 2023. doi:10.3389/fneur.2023.1075736

Plotas, P., Nanousi, V., Kantanis, A., Tsiamaki, E., Papadopoulos, A., Tsapara, A., et al. (2023). Speech deficits in multiple sclerosis: a narrative review of the existing literature. *European Journal of Medical Research* 28, 252. doi:10.1186/s40001-023-01230-3
